# The Promising Effect of Ascorbic Acid and Paracetamol as Anti-Biofilm and Anti-Virulence Agents against Resistant *Escherichia coli*

**DOI:** 10.3390/cimb46070406

**Published:** 2024-07-02

**Authors:** Sara M. Eltabey, Ali H. Ibrahim, Mahmoud M. Zaky, Adel Ehab Ibrahim, Yahya Bin Abdullah Alrashdi, Sami El Deeb, Moustafa M. Saleh

**Affiliations:** 1 Microbiology Program, Botany Department, Faculty of Science, Port Said University, Port Said 42521, Egypt; saraheltabay@sci.psu.edu.eg; 2 Botany Department, Faculty of Science, Port Said University, Port Said 42521, Egypt; aliibrahim@sci.psu.edu.eg (A.H.I.); zakymahmoud67@gmail.com (M.M.Z.); 3 Natural and Medical Sciences Research Center, University of Nizwa, P.O. Box 33, Birkat Al Mauz, Nizwa 616, Oman; adel@unizwa.edu.om; 4 College of Pharmacy and Nursing, University of Nizwa, Birkat Al Mauz, Nizwa 616, Oman; alrashdiyahya@unizwa.edu.om; 5 Institute of Medicinal and Pharmaceutical Chemistry, Technische Universitaet Braunschweig, 38106 Braunschweig, Germany; 6 Microbiology and Immunology Department, Faculty of Pharmacy, Port Said University, Port Said 42521, Egypt; mostafa.mohamed@pharm.psu.edu.eg

**Keywords:** antibiotic resistance, *E. coli*, anti-virulence, ascorbic acid, paracetamol, virulence inhibition

## Abstract

*Escherichia coli* is a major cause of serious infections, with antibiotic resistance rendering many treatments ineffective. Hence, novel strategies to combat this pathogen are needed. Anti-virulence therapy is a promising new approach for the subsequent era. Recent research has examined the impact of sub-inhibitory doses of ascorbic acid and paracetamol on *Escherichia coli* virulence factors. This study evaluated biofilm formation, protease production, motility behavior, serum resistance, expression of virulence-regulating genes (using RT-PCR), and survival rates in a mouse model. Ascorbic acid significantly reduced biofilm formation, protease production, motility, and serum resistance from 100% in untreated isolates to 22–89%, 10–89%, 2–57%, and 31–35% in treated isolates, respectively. Paracetamol also reduced these factors from 100% in untreated isolates to 16–76%, 1–43%, 16–38%, and 31–35%, respectively. Both drugs significantly down-regulated virulence-regulating genes *papC*, *fimH*, *ompT_m*, *stcE*, *fliC*, and *kpsMTII*. Mice treated with these drugs had a 100% survival rate compared with 60% in the positive control group control inoculated with untreated bacteria. This study highlights the potential of ascorbic acid and paracetamol as anti-virulence agents, suggesting their use as adjunct therapies alongside conventional antimicrobials or as alternative treatments for resistant *Escherichia coli* infections.

## 1. Introduction

*Escherichia coli* (*E. coli*) is an essential component of the normal gut microbiota in humans. While typically beneficial, *E. coli* can acquire various genes that transform it into a harmful pathogen capable of causing a range of diseases [1]. *E. coli* is the main reason for neonatal meningitis, with percentages ranging from 28 to 29%, and a death rate of 33% [2]. Moreover, it is the main reason for urinary tract infections (UTIs), which include uncomplicated and catheter-related UTIs. These in turn can lead to severe problems like pyelonephritis, possibly causing septic shock or death [3]. It is expected that most adult women, at least 50–60%, will get a UTI once in their life [4]. It is also known to cause bloody diarrhea, a condition called hemorrhagic colitis [1]. The prevalence of Hemolytic Uremic Syndrome (HUS) in individuals who have been exposed to *E. coli* varies between 5% and 15% [5]. Also, it is found in surgery wounds, and it is known to be one of the top bacteria from cultures of Surgical Site Infections with percentages ranging from 13.3% to 15.3% [6].

There is now growing concern around the world about the growth of multidrug resistance (MDR) in *E. coli*. Irrational antibiotic policies are to blame for the rise in antimicrobial resistance (AMR), mostly from the empirical prescription of broad-spectrum antibiotics without first conducting antibiotic susceptibility testing [7]. In 2019, AMR caused an estimated 4.95 million deaths worldwide. Predictions prove that by 2050, AMR might be the top cause of death all over the world. It is expected to kill about 10 million people each year [8]. Thus, new strategies to manage infections and expand treatment choices must be assessed [9,10].

The pathogenicity of *E. coli* is attributed to the production of many virulence factors, including biofilm development, protease production, motility, capsule protection, toxins secretions, iron acquisition, and serum resistance [11]. The virulence factors in *E. coli* are controlled by different virulence-regulating genes. For instance, *papC* and *fimH* have an important role in biofilm formation ability. Other genes, *ompT_m* and *StcE*, encode the synthesis of different bacterial proteases. The *fliC* gene encodes the production of a subunit protein of the flagella of *E. coli* for motility, and *kpsMTII* encodes the capsule formation that enhances *E. coli* pathogenesis [11,12,13,14,15,16].

Targeting the virulence factors of MDR bacteria is considered a next-generation treatment strategy, which is different from the conventional use of antibiotics in their intent to disable pathogens rather than kill them [17]. Repurposing some FDA-approved drugs is one of the most promising anti-virulence tactics to find off-label applications unrelated to their primary use [18]. This strategy overcomes two major problems of antibiotics including the development of resistance and the unintended killing of the normal flora. By the same token, the guarantee of the safety of repurposed medications reduces both the time and expenses of their development [19,20,21].

Ascorbic acid is a natural compound acting as a protective agent of the body against the free reactive oxygen species (ROS), and it has microbicidal action. Previously, ascorbic acid has shown a decreasing effect in the development of MRSA biofilms and inhibiting the varied virulence factors of *Pseudomonas aeruginosa* (*P. aeruginosa*) [22]. Paracetamol is widely used as an analgesic and antipyretic; in previous studies, it was proven to have an anti-virulence ability by obstructing the development of biofilm, protease, and various virulence factors produced by *P. aeruginosa* [23,24]. Such findings motivated and inspired us to investigate the potential anti-virulence effects of ascorbic acid and paracetamol against *E. coli*.

The outcomes of this research may enhance trials that aim to find an alternative approach to combat *E. coli* infections and reduce the frequency of AMR in this challenging pathogen.

This study offers an alternative strategy that involves the utilization of some available drugs in attenuating the virulence factors of MDR *E. coli* as potential anti-virulence agents for the management of *E. coli* infections to be used alone or in combination with antibiotics. To our knowledge, this is one of the first studies to investigate the effect of ascorbic acid and paracetamol as anti-virulence drugs on *E*. *coli* in Egypt.

## 2. Materials and Methods

### 2.1. Tested Isolates

In the present study, six clinical isolates of *E. coli* were tested (labeled as E1 to E6), which were obtained as a gift from the stock culture collection of the Department of Microbiology and Immunology, Faculty of Medicine, Suez Canal University. These isolates were previously obtained from patients with UTIs who were hospitalized at the Suez Canal University Specialized Hospital (Ismailia, Egypt). The isolates were previously identified by VITEK 2 COMPACT SYSTEM (Biomérieux, Craponne, France). The bacterial identification card VITEK^®^ ID GNB (Biomerieux, Craponne, France) was used in compliance with the manufacturer’s guidelines.

### 2.2. Antibiotic Susceptibility Testing

The antibiotic susceptibility profile of the tested six isolates was carried out by the disc diffusion method against eight antibiotics of different groups, including levofloxacin (LEV; Quinolones), meropenem (MRP; Carbapenems), cefotaxime (CTX; Cephalosporins), trimethoprim/sulfamethoxazole (SXT; Sulfonamides), amikacin (AK; Aminoglycosides), piperacillin (PRL; β-lactams), doxycycline (DO; Tetracyclines), and amoxicillin/clavulanic acid (AMC; β-lactams + β-lactamase inhibitor) (Oxoid, Basingstoke, UK). The tested isolates were resuscitated in tryptone soya broth (TSB) from Merck (Darmstadt, Germany) and incubated overnight. After incubation, the cultures of the tested isolates in TSB were diluted to reach (equal) the turbidity of 0.5 MacFarland standard, and an aliquot of 10 µL of each isolate was added to the surface of Mueller Hinton agar (MHA), (Merck, Darmstadt, Germany) and then distributed by swabbing perpendicularly on the MHA surface. The tested antimicrobial discs were put on the surface of MHA. After incubating overnight, the diameter of each inhibition zone around the discs was measured in mm, and the results were interpreted according to the Clinical and Laboratory Standards Institute (CLSI) [25].

### 2.3. Minimal Inhibitory Concentration (MIC) Assessment of Ascorbic Acid and Paracetamol

The determination of the MIC of ascorbic acid and paracetamol, which were obtained from IDI Pharmaceuticals (Port Said, Egypt), was carried out using the broth microdilution technique following the CLSI [25]. In brief, an overnight culture was diluted in fresh Luria Bertani (LB) broth (Merck, Darmstadt, Germany) to achieve a concentration of 0.5 McFarland standard. The tested drugs were subjected to 2-fold serial dilutions with varying concentrations (0.25, 0.5, 1, 2, 4, 8, 16, 32, and 64 mg/mL), and 100 μL of the resulting solutions were mixed with 100 μL of microbial suspensions in microtiter plates. After incubation for 24 h at 37 °C, the MIC values of the tested drugs were determined as the lowest concentration of the tested drugs that could inhibit the visible growth. The positive control was wells containing bacteria without tested drugs.

### 2.4. Assessment of the Ascorbic Acid and Paracetamol Effects on Bacterial Growth

To assess the impact of the sub-MICs of the tested drugs on the growth of *E. coli* over time, a growth curve experiment was performed. In brief, the overnight-tested isolates were resuscitated in LB broth and adjusted until an optical density (OD_600_) of 0.2 using a spectrofluorometer (Biotek, Shoreline, WA, USA). The cultures were then dispensed into three 500 mL Erlenmeyer flasks. The sub-MICs of ascorbic acid and paracetamol were put into two of the flasks, whereas the third flask was kept as an untreated control. Subsequently, the flasks were incubated under shaking conditions at 37 °C, and 1 mL of each culture was withdrawn immediately after the addition of the tested drugs (t_0_), as well as every thirty minutes for two hours. The OD_600_ was measured using a spectrofluorometer (Biotek, Shoreline, WA, USA) [17].

### 2.5. Virulence Factor Inhibition Assay

#### 2.5.1. Biofilm Inhibition Assay

The measurement of biofilm formation inhibition in the tested isolates was conducted as previously described [26]. Initially, a suspension of the tested isolates in TSB was prepared from an overnight culture and was set to 1 × 10^6^ CFU/mL. Subsequently, 200 μL of the bacterial suspension was inoculated into the wells of a microtiter plate in the presence and absence of 200 μL sub-MIC of the tested drugs, with subsequent incubation for 48 h at 37 °C. Then, the TSB was discarded, and the plates were washed with distilled water, followed by air drying. After 20 min of exposure to 200 μL of 99% fixing methanol, the biofilm was dyed for 15 min with 200 μL of 1% crystal violet solution. Following the cleansing of the plate, the crystal violet was dissolved in 33% glacial acetic acid, and the OD_570_ of the solubilized dye was measured using a spectrofluorometer (Biotek, Shoreline, WA, USA). The degree of biofilm inhibition was calculated using the following formula:

% of biofilm inhibition = (OD reading in absence of tested drug − OD reading in presence of tested drug)/OD reading in absence of tested drug)

The cut-off optical density (ODc), which is defined as three standard deviations above the mean OD of the negative control, was used for the classification of tested strains according to biofilm production. All the tested strains and the negative controls had their mean OD values determined. The negative control was TSB without bacteria. According to Stepanović et al., 2007 [26], the tested isolates were classified into non-biofilm producers (OD < ODc), weak biofilm producers (ODc < OD < 2 ODc), moderate biofilm producers (2 ODc < OD < 4 ODc), or strong biofilm producers (OD > 4 ODc).

#### 2.5.2. Proteases Inhibition Assay

The total protease assay was conducted using the modified skimmed milk method [27]. Briefly, the bacterial isolate overnight cultures were inoculated in LB broth to attain turbidity of the 0.5 McFarland standard in the presence and absence of the sub-MICs of ascorbic acid and paracetamol. Following centrifugation of the bacterial cultures at 10,000 rpm for 10 min, an aliquot of 200 μL of the culture supernatants was obtained and incubated with 1.0 mL skimmed milk (1.25% in sterile distilled water) at 37 °C for 30 min. Uninoculated tubes served as negative controls. Finally, the proteolytic activity was determined by measuring the OD_600_ of skimmed milk for treated and untreated isolates using a spectrophotometer (Biotek, Shoreline, WA, USA).

#### 2.5.3. Swimming Motility Inhibition Assay

The swimming motility assay was conducted following previously described procedures [28]. In brief, the overnight-tested isolates were resuscitated in LB broth until an OD_600_ of 0.2 in the presence and the absence of the sub-MICs of ascorbic acid and paracetamol. Then, 5 μL of both treated and untreated isolates were inoculated onto the center of a 0.4% soft LB agar plate, followed by incubation at 28 °C. Uninoculated plates served as a negative control. The bacterial colony fronts were monitored to determine the extent of swimming migration, and progress was measured after 24 h.

#### 2.5.4. Serum Resistance Inhibition Assay

The tested isolates were cultured overnight and then adjusted to 1 × 10^4^ CFU/mL in the presence and absence of the sub-MICs of ascorbic acid and paracetamol. A 50 μL aliquot of the diluted bacterial suspensions was added to 50 μL of fresh human serum and incubated for three hours. Subsequently, dilutions from all the tested isolates were plated onto LB plates and incubated overnight at 37 °C, after which, counts were determined. Uninoculated plates were used as a negative control. The survival rates of the bacterial suspensions were calculated as percentages resulting from the log reduction of the untreated surviving colony-forming unit (CFU) and treated surviving CFU [29].

#### 2.5.5. The Quantification of Gene Expression Levels of Virulence Factors Encoding Genes

The isolate E1 (representative isolate) was cultured overnight at 37 °C in TSB, with and without the sub-MICs of the tested drugs, until the bacteria reached the middle log phase (OD_600_ of 0.5–0.6). Following the manufacturer’s instructions, the pellets of the E1 isolate were obtained by centrifugation at 6000× *g* for 10 min, and RNA was purified using TRIzols Reagent (15596026, Life Technologies, Carlsbad, CA, USA). The virulence factors regulating genes *papC*, *fimH*, *ompT_m*, *stcE*, *fliC*, and *kpsMTII* were subjected to reverse transcription followed by qRT-PCR. This process was conducted in accordance with the procedures outlined in the QuantiTect Reverse Transcription kit (Qiagen, USA). The Rotor-Gene Q (Qiagen, Germantown, MD, USA) was utilized to perform the qRT-PCR analysis using the primers in Table 1. The housekeeping gene 16S rRNA was used to standardize the relative expression values of each gene and the results were calculated using the 2^−∆∆Ct^ method [30].

#### 2.5.6. In Vivo Mice Survival Test

In brief, *E. coli* cultures were incubated with and without the sub-MICs of the tested drugs and adjusted to 0.5 McFarland standard in phosphate-buffered saline (PBS). Five groups of albino mice (*Mus musculus*) were studied, each consisting of 10 three-week-old mice. Two negative control groups were used, one group was intraperitoneally injected in the lower left quadrant of the abdominal region with 100 μL of sterile PBS, and the second group was left uninoculated. A positive control group was injected with 100 μL of untreated *E. coli*. The remaining two groups were injected with 100 μL of the treated isolates. The survival rate was recorded for three consecutive days and plotted by the Kaplan–Meier method [34.

### 2.6. Statistical Analysis

Data analysis was performed using the GraphPad Prism 8.0.2 software package. The calculations were performed using the average values ± standard errors obtained from three biological experiments, each of which included three technical replicates. The impact of the examined drugs on *E. coli* virulence factors was assessed by using One-Way ANOVA according to Dunnett’s Multiple Comparison test at *p* < 0.05 or *p* < 0.001, * for significance.

## 3. Results

### 3.1. Identification, Antibiotic Susceptibility, and Resistance Profile of the Isolates

The tested isolates were confirmed as *E. coli* using the VITEK 2 COMPACT system (BIOMERIEUX, Craponne, France). All six isolates exhibited 100% resistance to AMC, PRL, CTX, and SXT. Moreover, four isolates (67%) showed resistance against LEV, while only two isolates (33%) were resistant to DO. Conversely, no resistance was detected against either AK or MRP. The overall findings of the antibiotic susceptibility tests are presented in Table 2.

### 3.2. MIC Values and the Effect of the Sub-MICs of Ascorbic Acid and Paracetamol on Bacterial Growth

It was found that 16 mg/mL of both ascorbic acid and paracetamol separately inhibited the growth of the tested *E. coli* isolates. The inhibitory activities of the tested drugs against *E. coli* virulence factors were conducted at a concentration of one-fourth MIC (4 mg/mL) as lower concentrations did not show any inhibitory impact on virulence factor production by phenotypic tests.

**Figure 1:**
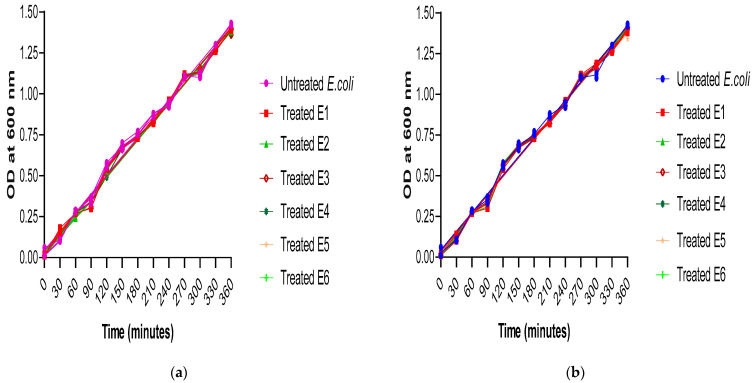
Comparison of the growth rate among treated and untreated *E. coli* isolates under the effect of (**a**) ascorbic acid and (**b**) paracetamol. The experiment was performed in triplicate. The statistical significance level was *p* < 0.001 using linear regression in GraphPad prism.

Regarding the growth curve, it was revealed that the sub-MICs of ascorbic acid and paracetamol showed no significant difference in the growth of *E. coli* isolates, either treated or untreated (Figure 1a,b). This indicates that any inhibitory effect of ascorbic acid and paracetamol on virulence factor production is not due to any adverse effect on the tested isolates’ viability.

### 3.3. The Influence of Ascorbic Acid and Paracetamol on E. coli Virulence Factors

#### 3.3.1. Biofilm Inhibition Assay

Ascorbic acid and paracetamol significantly reduced the biofilm formation ability of *E. coli*. Four untreated isolates were strong biofilm producers (E1, E3, E4, and E6), and two isolates were moderate biofilm producers (E2 and E5). After ascorbic acid treatment, two isolates were moderate biofilm producers (E1 and E4), and four isolates were weak biofilm producers (E2, E3, E5, and E6). After paracetamol treatment, two isolates became moderate biofilm producers (E1, E3), while the other four isolates were weak biofilm producers (E2, E4, E5, and E6).

**Figure 2:**
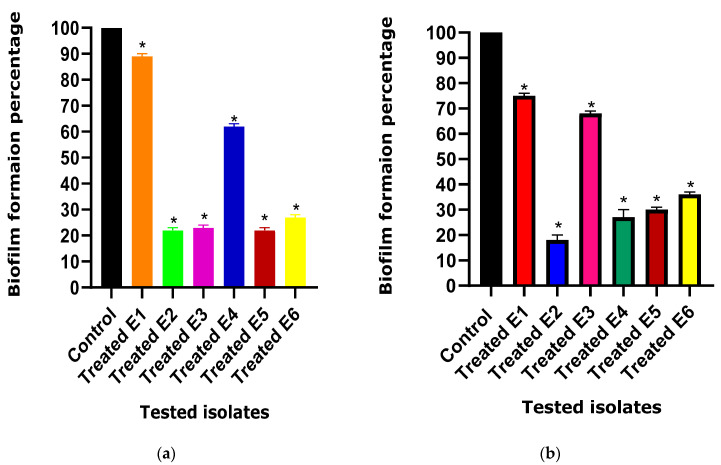
Anti-biofilm activity of (**a**) ascorbic acid and (**b**) paracetamol against *E. coli* isolates. The anti-biofilm activity between the treated and untreated (control) *E. coli* isolates under the effect of the tested drugs was compared. The results displayed represent the means ± standard errors. * Significance, *p* value < 0.05.

Biofilm formation was tested in the presence of a subinhibitory concentration of the tested drugs, and it was compared to the control (untreated isolates) in which no tested drugs were present. Remarkably, the biofilm formation ability exhibited a significant decrease from 100% in untreated isolates to percentages ranging from 22% to 89% and from 16% to 76% in the presence of ascorbic acid and paracetamol, respectively (Figure 2).

#### 3.3.2. Total Protease Inhibition Assessment

**Figure 3:**
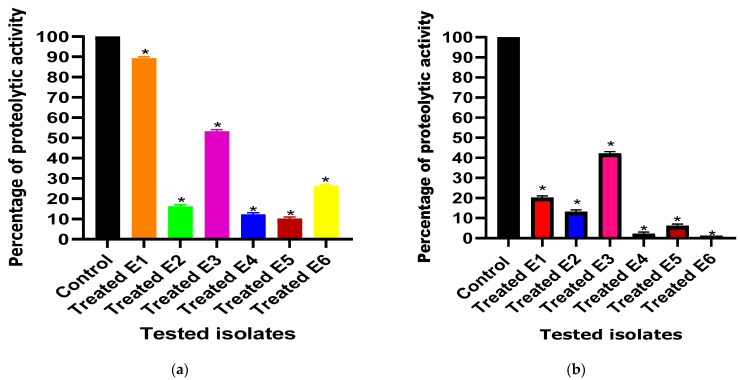
Effect of one-fourth MIC of (**a**) ascorbic acid and (**b**) paracetamol on proteolytic activity in *E. coli* isolates. The proteolytic activity among treated and untreated (control) *E. coli* isolates under the effect of the tested drugs was compared. The results displayed represent the means ± standard errors. * Significance, *p* value < 0.05.

In the present study, both ascorbic acid and paracetamol were found to significantly decrease the proteolytic activity from 100% in untreated isolates to percentages ranging from 10% to 89% and from 1% to 43% in ascorbic acid- and paracetamol-treated isolates, respectively (Figure 3).

#### 3.3.3. Swimming Motility Inhibition Assay

**Figure 4:**
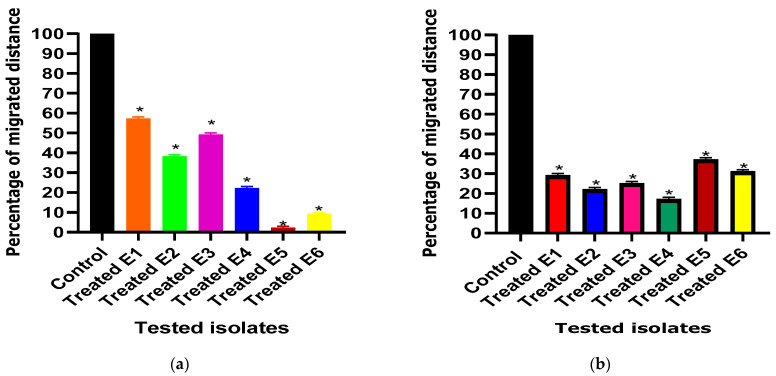
Effect of one-fourth MIC of (**a**) ascorbic acid and (**b**) paracetamol on the swimming motility of *E. coli* isolates. The swimming motility between the treated and untreated (control) *E. coli* isolates under the effect of the tested drugs was compared. The results displayed represent the means ± standard errors. * Significance, *p* value < 0.05.

Interestingly, the swimming motility behavior exhibited a significant decrease from 100% in untreated isolates to percentages ranging from 2% to 57% and from 16% to 38% in the presence of ascorbic acid and paracetamol, respectively (Figure 4).

#### 3.3.4. Serum Resistance Inhibition Assay

**Figure 5:**
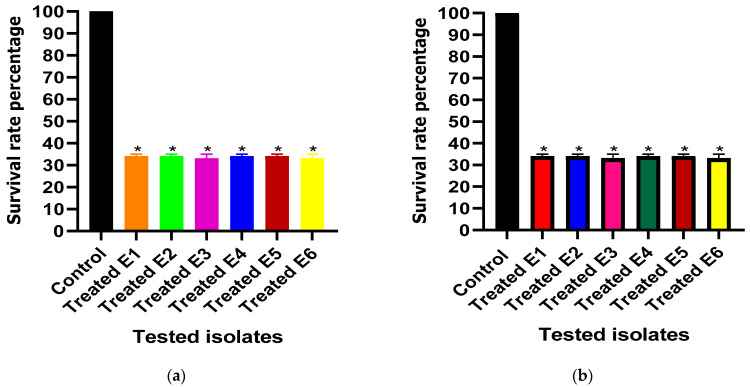
Effect of one-fourth MIC of (**a**) ascorbic acid and (**b**) paracetamol on serum resistance in *E. coli* isolates. The serum resistance between the treated and untreated (control) *E. coli* isolates under the effect of the tested drugs was compared. The results displayed represent the means ± standard errors. * Significance, *p* value < 0.05.

The administration of ascorbic acid and paracetamol at one-fourth MIC resulted in a significant reduction in serum resistance from 100% in untreated isolates to percentages ranging from 31% to 35% and from 31% to 35% in ascorbic acid- and paracetamol-treated isolates, respectively (Figure 5).

#### 3.3.5. The Relative Gene Expression Levels of Virulence Factor-Encoding Genes in *E. coli*

**Figure 6:**
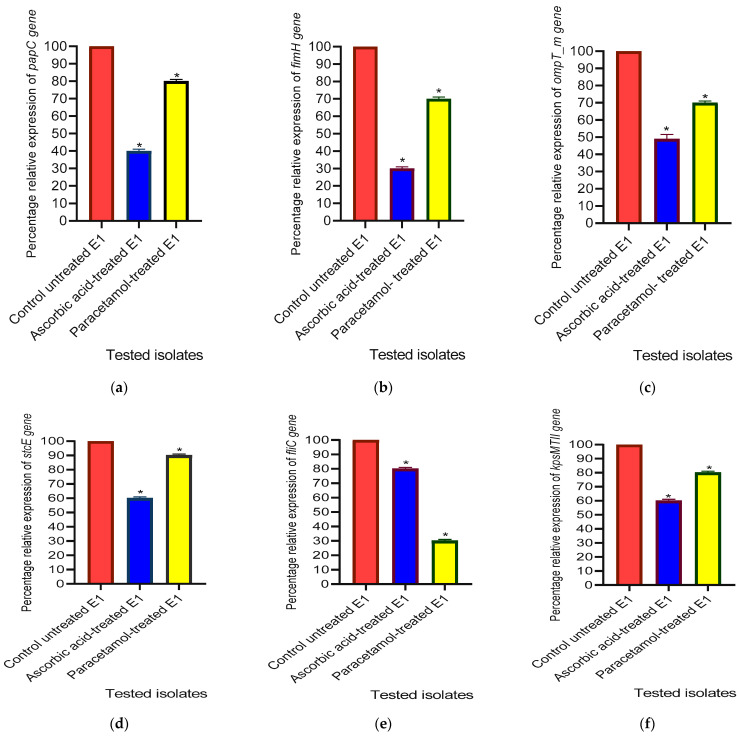
Effect of one-fourth MIC of ascorbic acid and paracetamol on virulence factor-regulating gene ((**a**) *papC*, (**b**) *fimH*, (**c**) *ompT_m*, (**d**) *stcE*, (**e**) *fliC* and (**f**) *kpsMTII*) in *E. coli* isolates. The data displayed represent the means ± standard errors. * Significance, *p* value < 0.05.

At sub-MIC, both ascorbic acid and paracetamol showed a 60% and 20% reduction in the relative expression level of the *papC* gene. Meanwhile, the expression level of *fimH* was suppressed by 70% and 30% in ascorbic acid- and paracetamol-treated isolates, respectively. Also, the expression level of the *ompT_m* gene was suppressed by 50% and 30% in ascorbic acid- and paracetamol-treated isolates, respectively. Furthermore, both ascorbic acid and paracetamol showed inhibitory effects on the relative expression levels of the *stcE* gene by 40% and 10%, respectively. Additionally, *fliC* relative expression levels were significantly lowered by 20% and 70% in ascorbic acid- and paracetamol-treated isolates, respectively, whereas the relative expression level of the *kpsMTII* gene was reduced by 40% in ascorbic acid-treated isolates and 20% in paracetamol-treated isolates (Figure 6).

#### 3.3.6. In Vivo Mice Survival Model

**Figure 7:**
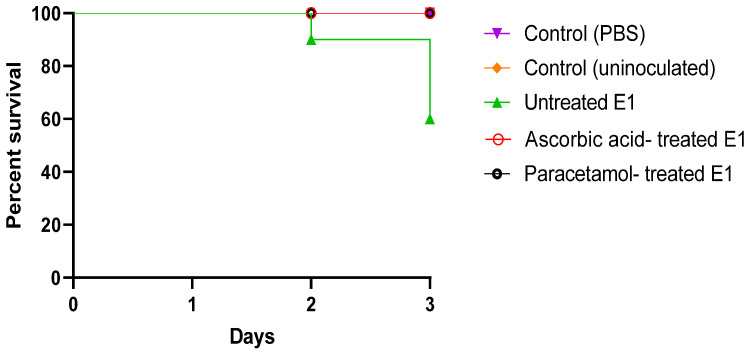
In vivo mice survival test of ascorbic acid- and paracetamol-treated isolates compared to untreated *E. coli* isolates plotted using the Kaplan–Meier method and calculated using the Log-rank test, with a significance of *p* ˂ 0.001.

Overall, 100% survival was observed in the negative control groups (uninoculated and PBS-injected mice). Only 60% of the mice injected with untreated bacteria in the positive control survived. Interestingly, mice injected with bacteria treated with sub-MIC doses of both ascorbic acid and paracetamol displayed a 100% survival rate (Figure 7).

## 4. Discussion

*E. coli* harbors varied virulence determinants including a wide range of weapons. Specifically, biofilm is attached to either biologic or non-biologic surfaces, serving to safeguard the microbes against unfavorable environmental conditions. The development of antibiotic resistance in biofilm structural components makes its eradication a significant challenge [35,36].

In the present study, the anti-biofilm and the anti-virulence properties of ascorbic acid and paracetamol were assessed against *E. coli.* Basically, the tested drugs exhibited significant anti-virulence activities after testing biofilm formation, protease production, motility behavior, and serum resistance.

It was observed that ascorbic acid demonstrated a capacity to hinder the production of biofilm, protease production, motility behavior, and serum resistance of *E. coli* from their initial value of 100% production in control untreated isolates to values within the range of 22–89%, 10–89%, 2–57%, and 31–35%, respectively. A previous study similarly found that ascorbic acid led to a decrease in biofilm formation and protease production, with reduction percentages ranging from 1.35% to 41.77% and 44% to 75%, respectively, in *S. aureus* [17]. In another study, ascorbic acid exhibited a decrease in biofilm formation ability in *Stenotrophomonas maltophilia*, which is in accordance with our results [37]. Also, ascorbic acid was found to inhibit biofilm formation and motility in *P. aeruginosa* [38]. Similarly, in a previous study, ascorbic acid was shown to reduce certain virulence traits in *P. aeruginosa*, such as protease activity, biofilm formation, and motility, with reduction percentages ranging from 80% to 85%, from 55% to 64%, and from 60% to 72%, respectively [39]. In a similar manner, previous research stated that ascorbic acid inhibited the biofilm formation and capsule formation that is responsible for serum resistance in *Klebsiella pneumoniae* (*K. pneumoniae*) [40].

The mechanism of ascorbic acid anti-virulence activities may be attributed to its capability to disrupt the bacterial membrane proton motive force driving efflux activities. Consequently, the transportation of exopolysaccharides and capsule polysaccharides was impeded, leading to the inhibition of biofilm formation and capsule synthesis and its ability to inhibit some virulence-regulating genes [40].

In the present research, paracetamol impeded the formation of biofilm, production of proteases, and mobility of *E. coli* isolates. Similarly, in previous research, paracetamol inhibited the biofilm formation ability of *P. aeruginosa* by 68.15% [24]. Paracetamol was found to inhibit biofilm formation, proteases, and motility by percentages of 39.7–93%, 8.7%, and 7.7–29.4%, respectively, in *Acinetobacter baumannii* [41]. In an earlier study, paracetamol curtailed a significant degree of biofilm development in *P. aeruginosa* and *Staphylococcus epidermidis* (*S. epidermidis*) [42]. In accordance with the proposed results, paracetamol lowered biofilm development by 100% in *S. epidermidis* [43].

The anti-virulence impact of paracetamol can be attributed to the disturbance of the polymeric biofilm matrix and a reduction in the associated virulence factors. This results in rendering the bacteria powerless and defenseless, knocking down the resistance, because of the reduced selective pressure that is exerted on the bacteria [44].

At the molecular level, the relative expression levels of the virulence-regulating genes, *papC*, *fimH*, *ompT_m*, *stcE*, *fliC*, and *kpsMTII*, were decreased by ascorbic acid and paracetamol. In the present investigation, the examined genes exhibited a decline in relative gene expression when subjected to the influence of ascorbic acid. Ascorbic acid demonstrated its capability to suppress certain genes associated with virulence involved in biofilm formation and polysaccharide capsule production in *K. pneumonia* [40]. Additionally, an earlier study found that ascorbic acid impeded the expression of specific virulence-related genes in *S. aureus* [17]. Furthermore, ascorbic acid was revealed to hinder the activity of genes related to biofilm formation and antibiotic resistance in *P. aeruginosa* [45]. Gene downregulation associated with biofilm formation in *Stenotrophomonas maltophilia* by ascorbic acid was also proved [37]. Similarly, a previous report showed that ascorbic acid suppressed the virulence-regulating genes of *P. aeruginosa* at the molecular level [39].

On the other hand, paracetamol downregulated the virulence factor regulatory genes of *S. aureus* by percentages of between 1 and 34% in a recent study by Ozturk et al., which is in accordance with the proposed results [46]. The anti-virulence impact of paracetamol may be attributed to the role of interference with specific proteins such as AbaR proteins in the bacterial cell that control receiving the Acyl homoserine lactone (AHL) signal of quorum sensing, which regulates virulence factor production [41].

Considering the present study, it was deemed essential to characterize *E. coli* pathogenesis in vivo. Interestingly, both drugs under study exhibited 100% protection of the mice injected with treated bacteria, confirming our phenotypic and genotypic results. The findings were consistent with those of a recent study [17], which revealed that ascorbic acid decreased the pathogenicity of *S. aureus* in vivo, impacting biofilm and protease production. Similarly, a previous study demonstrated that ascorbic acid mitigated *S. aureus* virulence factors in a mouse survival test [22]. Notably, the efficacy of ascorbic acid in attenuating the virulence traits of *P. aeruginosa* in vivo, including anti-biofilm properties has been reported recently by Aldawsari et al. [47]. Ascorbic acid had an anti-biofilm effect on a mouse model infected with *P. aeruginosa* [45]. Moreover, ascorbic acid was able to lower virulence traits in *K. pneumonia*, increasing mouse survival [40]. Furthermore, Ahsan et al. noted that paracetamol exhibited a reducing impact on the pathogenesis of *P. aeruginosa* [48].

## 5. Conclusions

According to the results of the present study, it can be concluded that both ascorbic acid and paracetamol have shown promising efficacy in inhibiting biofilm formation and the production of virulence factors in *E. coli*. These results suggest that ascorbic acid and paracetamol could be used as an alternative therapy in the treatment of MDR *E. coli*, either individually or in combination with conventional antibiotics as a novel approach to overcome the AMR issue.

## Figures and Tables

**Table 1:** Primers of the tested virulence genes.

Genes	Primers	References
*16SrRNA*	F */AGT TTG ATC MTG GCT CAG	[31]
	R */GGA CTA CHA GGG TAT CTA AT	
*papC*	F/TGA TAT CAC GCA GTC AGT AGC	[32]
	R/CCG GCC ATA TTC ACA TAAC	
*fimH*	F/TGC AGA ACG GAT AAG CCG TGG	[32]
	R/GCA GTC ACC TGC CCT CCG GTA	
*ompT_m*	F/TTT GAT GCC CCA GAT ATC TAT CGG	[33]
	R/GGC TTT CCT GAT ATC CGG CCA TG	
*stcE*	F/AAG GGC CCC TCT GAG GTG TCT GTTAAA CCC GTG G	[12]
	R/AAA AA TGG CCA CGA AGT GGCCGC ACC GTC TCA GG	
*fliC*	F/ACA GCC TCT CGC TGA TCA CTC AAA	[13]
	R/GCG CTG TTA ATA CGC AAG CCA GAA	
*kpsMTII*	F/GCG CAT TTG CTG ATA CTG TTG	[11]
	R/CAT CCA GAC GAT AAG CAT GAGCA	

* F = forward, R = reverse.

**Table 2:** Antimicrobial susceptibility profile of the tested isolates.

	Antibiotics	LEV ^1^ 5 µg	MRP ^1^ 10 µg	CTX ^1^ 30 µg	SXT ^1^ 25 µg	AK ^1^ 30 µg	PRL ^1^ 100 µg	DO ^1^ 30 µg	AMC ^1^ 30 µg
Isolates									
E1 *		R ^2^	S ^2^	R	R	S	R	R	R
E2		R	S	R	R	S	R	S	R
E3		S	S	R	R	S	R	S	R
E4		S	S	R	R	S	R	R	R
E5		R	S	R	R	S	R	S	R
E6		R	S	R	R	S	R	S	R

^1^ LEV (levofloxacin), MRP (meropenem), CTX (cefotaxime), SXT (trimethoprim/sulfamethoxazole), AK (amikacin), PRL (piperacillin), DO (doxycycline), AMC (amoxicillin/clavulanic acid). ^2^ R (resistant), S (susceptible), E1 * (*E. coli* isolate number 1).

## Data Availability

The data are available upon request.
